# Early Neurodevelopmental Outcome of Very Low Birthweight Neonates with Culture-positive Blood Stream Infection: A Prospective Cohort Study

**DOI:** 10.7759/cureus.3492

**Published:** 2018-10-24

**Authors:** Laxman Singh, Sarthak Das, Vishnu B Bhat, Nishad Plakkal

**Affiliations:** 1 Paediatrics, Jawaharlal Institute of Postgraduate Medical Education and Research, Puducherry, IND; 2 Neonatology, Jawaharlal Institute of Postgraduate Medical Education and Research, Puducherry, IND

**Keywords:** culture positive sepsis, neurodevelopment follow up, outcome, vlbw neonate

## Abstract

Aim

Our study aimed to evaluate the early neurodevelopmental outcomes of very low birthweight (VLBW) neonates with culture-proven sepsis compared with VLBW neonates without sepsis.

Materials and methods

Our study included 80 VLBW neonates with blood culture-proven sepsis and 80 VLBW neonates without sepsis. For each neonate with sepsis, a control neonate of matching gender and gestational age was selected. We evaluated weight, length, and head circumference and conducted Hammersmith neonatal neurological examinations for each participant. We also conducted Developmental Assessment Screening II and vision and hearing impairment screening during a follow-up evaluation.

Results

We noted a developmental delay in 11 (23.4%) neonates with sepsis and two (3%) without sepsis at six months of age. The median developmental quotient, motor quotient, and mental quotients were significantly lower in the neonates with sepsis compared to those without sepsis.

Conclusions

Our findings suggest that sepsis significantly affects mortality and neurodevelopmental outcomes at six months in VLBW neonates. Therefore, preventive measures for sepsis in VLBW neonates is crucial, and those with culture-positive sepsis will require close monitoring and follow-up evaluations.

## Introduction

Survival among very low birth weight (VLBW) infants has improved due to advanced care via neonatal intensive care units (NICU). The incidence of VLBW births is a major public health problem with significant medical and financial impact due to the concomitant increase in long-term comorbidities such as poor neurodevelopmental outcomes. Major neurodevelopmental abnormalities among VLBW survivors include cerebral palsy, mental retardation, and visual and auditory deficits [1-2]. Hence, it is essential to identify the risk factors for poor neurodevelopmental outcomes so that interventions can be targeted to prevent or modify the risk factors. Because sepsis is a common issue among neonates admitted to the NICU, an understanding of the neurodevelopmental outcomes is also important for prognostication.

Neonates are at the highest risk for bacterial sepsis, with a prevalence of 1 to 10 per 1000 live births [1]. In neonates, sepsis is associated with high mortality and morbidity. Around 3.8 million neonatal deaths occur annually worldwide, which accounts for 42% of under-five mortality. Sepsis among VLBW infants affects morbidity and mortality, especially in a developing country like India [3]. Most VLBW neonates get at least one infection during their hospital stay due to a combination of an immature immune system, increased interventions like central venous catheterization and total parenteral nutrition, blood product transfusion, mechanical ventilation, and surfactant administration [1-2].

Studies from high-income countries identified sepsis as an important risk factor for adverse neurodevelopmental outcomes in VLBW infants. Because of the paucity of data in middle- and low-income countries, we conducted this study to record growth and neurodevelopmental abnormalities among neonates with sepsis.

## Materials and methods

We conducted this prospective cohort study from February 2015 to July 2016 in the NICU of the Jawaharlal Institute of Postgraduate Medical Education and Research, a tertiary-care hospital in Puducherry, India. VLBW was defined as birthweight below 1,500 g. All VLBW neonates with blood culture-proven sepsis were included in the study. For every neonate with sepsis, a gender and gestational age-matched neonate without sepsis was selected as the control. Infants who expired within 72 hours of admission or with major congenital malformations, genetic syndromes, or meningitis were excluded.

Based on previous studies, assuming a survival without neurosensory impairment of 55% in the study group and 75% among controls, 80 VLBW neonates with sepsis and 80 controls were calculated for an α of 0.05 (type I error; two-sided testing) and power of 80% [2,4-5]. We collected the study data prospectively, including the mothers’ demographics, underlying illnesses, medications administered, procedures administered to the infants, and complications during the hospital stay such as necrotizing enterocolitis (NEC), intraventricular hemorrhage (IVH; the highest grade documented), bronchopulmonary dysplasia (BPD), and retinopathy of prematurity (ROP).

We included a follow-up evaluation when the infants reached six months of age after discharge. We measured the weight, length, and head circumference to assess the growth during the follow-up evaluation. We assessed the motor outcomes using the Hammersmith Motor Function Scale at one month of age and measured the neurodevelopmental outcomes using the Differential Ability Scales, Second Edition (DASII). We also assessed the infants’ vision and hearing.

The DASII was used in our study for developmental assessment. From birth to 30 months of age, it estimates motor development and mental development as a Motor Developmental Quotient (DQ) and Mental DQ, respectively. Even the use of DASII in literature is scarce, yet it is commonly used in India [6-8]. A DASII score of less than 70 indicates significantly delayed development. Head circumference, length, and weight parameters were compared using the Fenton’s chart [9]. At age six months, we compared the growth parameters using the standard World Health Organization (WHO) growth charts for age and sex.

Statistical analysis

Statistical analysis was performed using SPSS for Windows, Version 15.0 (SPSS Inc., Chicago, IL). Continuous variables are presented as mean and standard deviations and were compared across groups using the Student’s *t*-test. If the continuous variable did not follow the normal distribution, we presented it as median with interquartile range and compared it using the non-parametric Mann-Whitney U test. The categorical outcomes were expressed as percentages and compared across groups using Chi-square or Fisher’s exact *t*-tests as appropriate. Results were considered statistically significant if *p* < 0.05.

## Results

During their NICU stay, 31 neonates with culture-positive sepsis (38.8%) and 13 neonates without sepsis (16.3%) died. Two neonates with sepsis (2.5%) and one without sepsis (1.3%) died after discharge. Forty-seven neonates with sepsis and 66 neonates without sepsis were included in the final analysis and assessed for long-term neurodevelopmental outcomes at six months of corrected age (Table 1).

**Table 1:** Comparison of baseline characteristics among two groups *Fisher's exact *t*-test,**Chi-square test SVD: spontaneous vaginal delivery; LSCS: lower (uterine) segment cesarean section; BMV: bag and mask ventilation; IVH: intraventricular hemorrhage; NEC: necrotizing enterocolitis; MV: mechanical ventilation; ROP: retinopathy of prematurity; OAE: otoacoustic emission test

Gender	Blood Culture *n *(%)		Total (%) (*n *= 160)	*P*	
	Positive (*n *= 80)	Negative (*n *= 80)			
Male	31 (38.8%)	33 (41.3%)	64 (40%)	0.747	
Gestational age (in completed weeks)							
<32	40 (50%)	40 (50%)	80 (50%)	1	
32-36	37 (46.3%)	38 (47.5%)	75 (46.9%)		
34-36	7 (8.8%)	8 (10%)	15 (9.4%)		
36-38	3 (3.8%)	2 (2.5%)	5 (3.1%)		
Birth weight (g)					
1000-1200	37 (46.3%)	25 (31.3%)	62 (38.8%)	0.003	
1201-1400	29 (36.3%)	30 (37.5%)	59 (36.9%)		
1401-1500	14 (17.5%)	25 (31.3%)	39 (24.4%)		
Mode of delivery					
SVD	61 (76.3%)	54 (67.5%)	115 (71.9%)		
LSCS	9 (11.3%)	15 (18.8%)	24 (15%)		
Instrumental delivery	10 (12.5%)	11 (13.7%)	21 (13.1%)		
Resuscitation details					
Initial steps only	53 (66.3%)	61 (76.3%)	114 (71.3%)		
BMV	15 (18.8%)	13 (16.3%)	28 (17.5%)		
Intubation	12 (15%)	4 (5%)	16 (10%)		
Chest compression	0 (0%)	2 (2.5%)	2 (1.3%)		
Apgar score					
				0.022*	
5 minutes	7.83 ± 1.20	8.35 ± 0.93	8.09 ± 1.10		
Positive Sepsis Screen	52 (65%)	32 (40%)	84 (52.5%)	0.002**	
IVH	7 (8.7%)	5 (6.2%)	12 (14.9%)	0.848	
NEC	35 (43.8%)	16 (20%)	51 (31.9%)	0.001	
Duration of MV (in days)					
0 to 6	22 (47.8%)	15 (71.5%)	37 (55.2%)		
7 to 12	19 (41.3%)	6 (28.6%)	25 (37.3%)	<0.001*	
ROP	16 (20%)	14 (17.5%)	30 (18.8%)	0.497	
Final outcome					
Survival	47 (58.8%)	66 (82.5%)	113 (70.6%)	0.002	
Weight at discharge (percentile)				0.023*	
<3^rd^	14 (17.5%)	16 (20%)	30 (18.8%)		
3^rd^ to 10^th^	7 (8.8%)	11 (13.8%)	18 (11.3%)		
10^th^ to 90^th^	26 (32.5%)	39 (48.8%)	65 (40.7%)		
Length at discharge (percentile)					
<3^rd ^	11 (13.8%)	10 (12.5%)	21 (13.1%)		
3^rd^ to 10^th^	15 (18.8%)	30 (37.5%)	45 (28.1%)	0.044*	
10^th^ to 90^th^	27 (33.8%)	26 (32.6%)	53 (33.1%)		
Head circumference at discharge (percentile)					
<3rd	0 (0%)	1 (1.3%)	1 (0.6%)		
3^rd^ to 10^th^	11 (13.8%)	14 (17.5%)	25 (15.6%)	0.032*	
10^th^ to 90^th^	38 (47.6.%)	51 (63.8%)	89 (28.8%)		
OAE					
Normal	78 (97.5%)	79 (98.8%)	157 (98.1%)	1	
Abnormal	2 (2.5%)	1 (1.3%)	3 (1.9%)		

The gestational ages of the infants were similar in the sepsis and non-sepsis groups, while the mean birth weight was lower in the sepsis group. Infants in the sepsis group also had lower Apgar scores than those in the non-sepsis group.

The most common organisms found were gram-negative bacteria (*n* = 53; 66.2%) followed by fungal organisms (*n* = 11; 13.8%), and gram-positive bacteria (*n* = 10; 12.5%) in the culture-positive neonates. *Klebsiella pneumoniae* was the most common type of bacterium found (33.8%) followed by *Escherichia coli* (15%), *Candida *species (13.8%), coagulase-negative *Staphylococci* (7.5%), *Acinetobacter* (6.2%), *Staphylococcus aureus* (5%), *Enterococcus* (5%), and *Pseudomonas* (3.8%).

As noted in Table 1, the mean duration of ventilation, occurrence of intraventricular hemorrhage, and NEC were significantly higher in the culture-positive group of infants.

BPD was observed in two infants (2.5%) with culture-positive sepsis. Thirty infants (18.8%) developed ROP, 16 of which were culture-positive (20%) and 14 culture negative (17.5%). The incidence of ROP and abnormal brainstem-evoked response audiometry (BERA) were similar for both groups in our study.

The mean birthweight (*P* = 0.023), length (*P* = 0.044), and head circumference (*P* = 0.032) were significantly lower in the culture-positive group compared with the control group. The six-month follow-up weight (*P* = 0.004), length (*P* ≤ 0.001), and head circumference (*P* = 0.001) were significantly lower in the culture-positive group compared with the control group (Table 2).

**Table 2:** Outcomes among the study population at six months of age *Fisher's exact *t*-test,**Chi-square test HC: head circumference; DASII, Differential Ability Scales, Second Edition.

	Blood culture *n *(%)		Total *n *(%)N = 113	*P*
	Positive (n = 47)	Negative (*n *= 66)		
Weight at six months (percentile)				
<3^rd^	16 (20%)	17 (21.3%)	33 (20.6%)	
3^rd^ to 10^th^	23 (28.8%)	26 (32.5%)	49 (30.6%)	0.004*
10^th^ to 90^th^	8 (10%)	23 (28.8%)	31 (19.4%)	
Length at six months (percentile)				
<3^rd^	17 (21.3%)	7 (8.8%)	24 (15%)	<0.001**
3^rd ^to 10^th^	22 (27.5%)	41 (51.3%)	63 (39.4%)	
10^th^ to 90^th^	8 (10.1%)	18 (22.5%)	26 (16.3%)	
HC at six months (percentile)				
<3rd	10 (12.5%)	7 (8.8%)	17 (10.6%)	0.001**
3^rd ^to 10^th^	16 (20%)	14 (17.5%)	30 (18.8%)	
10^th ^to 90^th^	21 (26.3%)	45 (56.3%)	66 (41.3%)	
Neurodevelopmental impairment				
Yes	11 (23.4%)	2 (3%)	13 (11.5%)	0.001**
Comparison of DASII scale				
Mental age	4.73 ± 0.79	5.14 ± 0.56	4.97 ± 0.69	0.002**
Mental quotient	78.89 ± 13.13	85.61±9.39	82.81 ± 11.54	0.002**
Motor age	4.48 ± 0.81	4.93 ± 0.74	4.74 ± 0.80	0.002**
Motor quotient	74.56 ± 13.43	83.22 ± 10.07	79.62±12.3	<0.001**
Developmental age	4.61 ± 0.77	5.07 ± 0.57	4.88 ± 0.70	<0.001**
Developmental quotient	77.41 ± 12.49	84.41 ± 9.21	81.5 ± 11.20	0.001**

**Figure 1:**
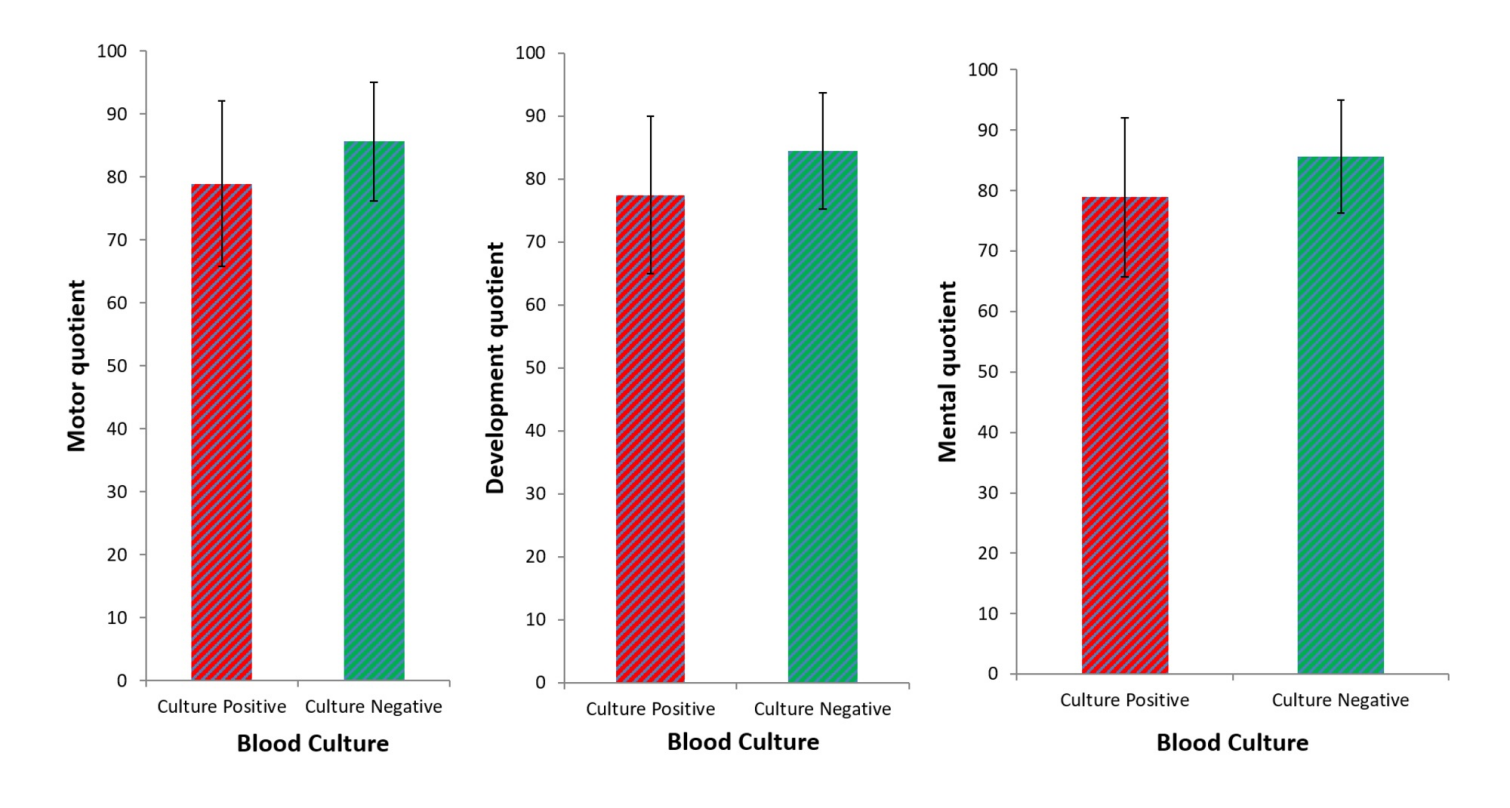
Comparison of the DASII scale among groups DASII: Differential Ability Scales, Second Edition

At the six-month follow-up, we noted a statistically significant difference (*P* = 0.001) in neurodevelopmental impairment (NDI) between the culture-positive infants and the controls. NDI occurred in 11 infants with culture-positive sepsis (23.4%) and two infants in the control group (3%). We also noted statistically significantly lower median developmental (*P* = 0.001), motor (*P* ≤ 0.001), and mental (*P* = 0.002) quotients in infants with culture-positive sepsis compared to controls (Table 2, Figure 1).

## Discussion

This study explores the effect of culture-proven bloodstream infection on adverse neurodevelopmental sequelae among VLBW infants. We found that the weight, length, and head circumference were significantly lower at six months of follow-up in infants with culture-positive sepsis compared to controls, which is very similar to reports in the literature [2-4,10]. Underdeveloped head circumference correlates with lower cognitive function later in life, implying an association between the postnatal growth and neurodevelopmental outcomes [10]. According to our findings, neonates with culture-positive sepsis are significantly more likely to have impaired head growth in later in life.

The incidence of BPD was not statistically significant between the groups in our study. The low incidence of BPD in our study may be because the infants enrolled in our study had lower potential risk factors for BPD (e.g., many were born extremely premature, managed with continuous positive airway pressure alone, had short duration of ventilation, had early administration of surfactant, lower target of oxygen saturation [90% to 95%], and higher mortality rate). A recent study on an extremely premature cohort and a report from the National Institute of Child Health and Human Development Neonatal Research Network reported high incidences of BPD, ranging from 40% to 60% [3]. Anderson et al. and Singer et al. reported BPD was an independent risk factor for poor NDI, contrasting with the report by Trittmann et al. that BPD was not a risk factor for NDI [11-13].

The high incidence of NEC we noted in the culture-positive group aligns with reports from Klinger et al. and Lee et al. [14-15]. However, Klinger et al. suggested that sepsis is not a risk factor for ROP, which contrasts with our findings. The difference may be due to the lower survival rate of infants with sepsis, differences in the study population, or due to the other potential confounders for ROP (e.g., prematurity and mechanical ventilation requirements).

Stoll et al. suggested hearing defects were more common in infants with culture-positive sepsis with gram-negative organisms or multiple organisms treated with a prolonged regimen of aminoglycosides, which contrasts with our findings [2].

The median developmental, motor, and mental quotients were significantly lower in VLBW infants with culture-positive sepsis compared with controls, and we noted a significantly higher NDI in culture-proven sepsis infants compared with controls, which align with the results of previous studies [5,16-17].

In our study, more neonates in the culture-positive group had poor neurodevelopmental outcomes later in life compared to the culture-negative control neonates. Gram-negative organisms were the most common organisms found in blood culture-positive neonatal sepsis cases. Schlapbach et al. reported more neonates with gram-positive sepsis developed cerebral palsy compared with uninfected neonates [5]. The incidence of neurodevelopmental impairment in our study was low compared to the reports from Stoll et al. and Schlapbach et al. [2,5]. The difference in findings may be due to the difference in the inclusion criteria (e.g., gestational age and birth weight) and important confounders of NDI like BPD and IVH. Schlapbach et al. indicated sepsis was one of the four major risk factors affecting the long-term outcomes; the other risk factors were BPD, brain injury, and ROP [5].

Extreme prematurity, low birth weights, intraventricular hemorrhage, and BPD are the non-modifiable risk factors which interfere with brain development and subsequent neurodevelopment. Sepsis is a preventable condition. Therefore, institutional protocols, such as minimizing central venous catheter use, prompt removal of catheters when no longer necessary, skin care, reduction of NEC by early administration of trophic feeds, weaning protocols, and early diagnosis and management of sepsis are important for better neurodevelopmental outcomes in VLBW infants.

Limitations

Our study was limited in that the neurodevelopmental and growth status were assessed at up to six months of corrected age only. Long-term follow-up until at least school age is required to determine the impact of sepsis on the behavioral and cognitive outcomes.

## Conclusions

We found a statistically significant difference in mortality and neurodevelopmental outcomes at six months of the corrected age between VLBW neonates with culture-positive sepsis and neonates without sepsis. Neonatal sepsis significantly prolonged the duration of mechanical ventilation and length of NICU stay, and it is an independent risk factor for ROP and NEC development. Apart from neurodevelopmental impairment, neonatal sepsis impairs growth later in life. Mortality and poor neurodevelopmental outcomes were high among the VLBW infants with positive cultures compared to those without sepsis. Hence, preventive measures to inhibit sepsis development in VLBW neonates are crucial, and those with culture-positive sepsis will require close monitoring and follow-up evaluations.
